# Titanium oxo/alkoxo clusters with both phosphonate and methacrylate ligands

**DOI:** 10.1007/s00706-015-1444-5

**Published:** 2015-04-08

**Authors:** Matthias Czakler, Christine Artner, Ulrich Schubert

**Affiliations:** Institute of Materials Chemistry, Vienna University of Technology, Vienna, Austria

**Keywords:** Titanium alkoxides, Methacrylate ligands, Phosphonate ligands, Structure analysis

## Abstract

**Abstract:**

The clusters Ti_5_O(O*i*Pr)_11_(OMc)(O_3_PR)_3_ (OMc = methacrylate; R = Et, CH_2_CH_2_CH_2_Br) and Ti_10_(O*i*Pr)_16_(OMc)_4_(O_3_PCH_2_CH=CH_2_)_10_ were obtained when Ti(O*i*Pr)_4_ was reacted with the corresponding bis(trimethylsilyl) phosphonate and methacrylic acid. Oxo clusters of the composition Ti_6_O_4_(O*i*Pr)_10_(OMc)_2_(O_3_PR)_2_, with a variety of groups R (Et, Ph, CH=CH_2_, CH_2_Ph, CH_2_CH=CH_2_, CH_2_CH_2_CH_2_Br, CH_2_CH_2_CN, CH_2_C(O)Me, CH_2_CH_2_OC(O)C(Me)=CH_2_), were formed instead, when a stoichiometric amount of water was added to the reaction mixture.

**Graphical abstract:**

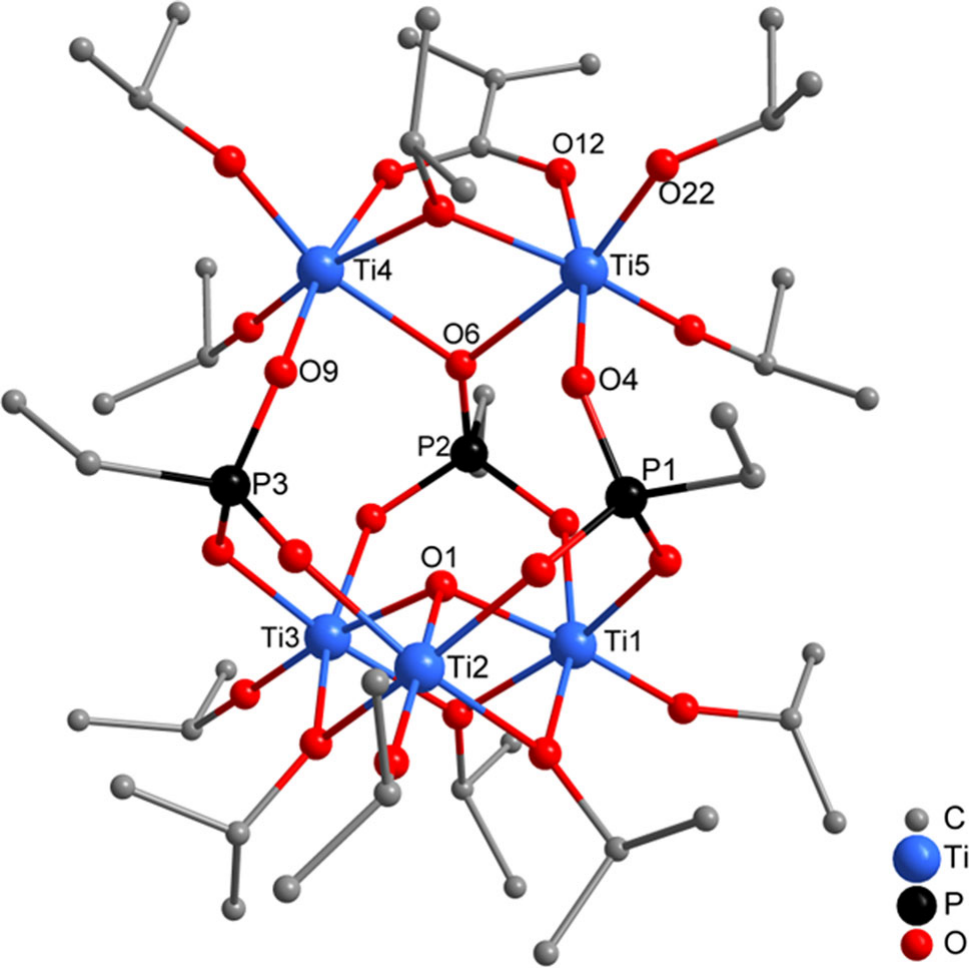

## Introduction

We have recently obtained phosphonate/acetate-substituted titanium oxo/alkoxo clusters from Ti(O*i*Pr)_4_ and bis(trimethylsilyl) phosphonates in the presence of acetic acid (AcOH), which served for in situ water generation through ester formation with eliminated *i*PrOH. Oxo clusters of the composition Ti_6_O_4_(O*i*Pr)_10_(OAc)_2_(O_3_PR)_2_ were obtained with a large variety of functional and non-functional substituents R (Et, CH_2_Ph, CH_2_C_10_H_7_, CH=CH_2_, CH_2_CH=CH_2_, CH_2_CH_2_CH_2_Cl, CH_2_CH_2_CH_2_Br), and also when the reaction conditions were varied [1]. This cluster type, which is also retained in solution, therefore appears to be very robust. Other clusters were only obtained in two exceptional cases (see below).

We extended these investigations by using methacrylic acid (McOH) instead of acetic acid. Methacrylic acid could also produce water through in situ ester formation, but would additionally provide reactive ligands in the obtained clusters and thus allow incorporating such clusters in organic polymers by polymerization with organic co-monomers (see review articles on cluster-crosslinked polymers [2, 3]). Especially the combination of ligands with different organic functionalities in one cluster appeared attractive. In this article, we report the outcome of these reactions.

## Results and discussion

**Fig. 1:**
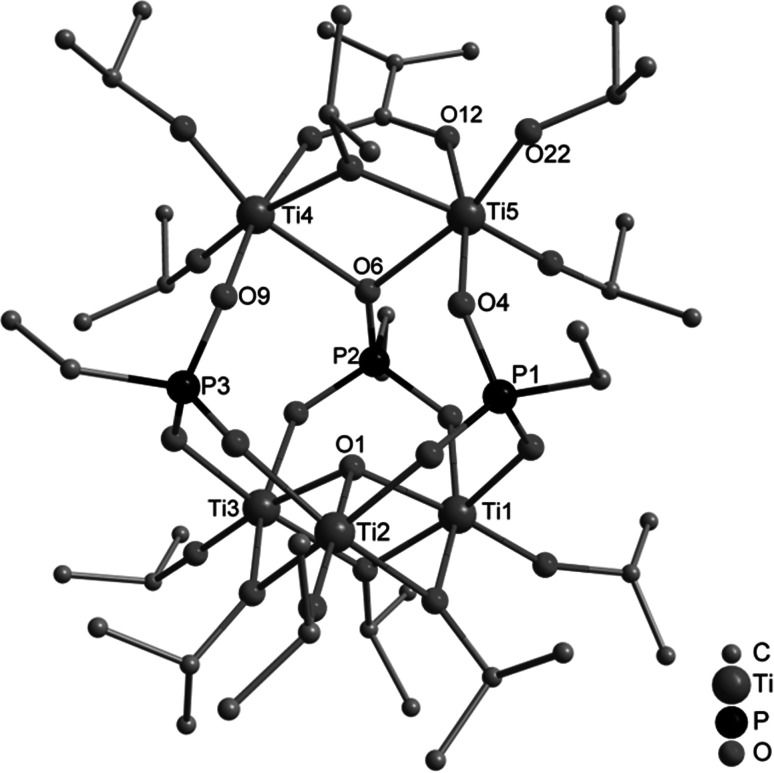
Molecular structure of Ti_5_(µ_3_–O)(µ_2_–O*i*Pr)_4_(O*i*Pr)_7_(OMc)(O_3_PEt)_3_ (**1**). Hydrogen atoms are omitted for clarity. Selected bond lengths/pm and angles/°: Ti(1)–O(1) 195.98(19), Ti(1)–O(3) 196.97(19), Ti(1)–O(13) 202.08(19), Ti(1)–O(17) 178.0(2), Ti(2)–O(1) 194.0(2), Ti(2)–O(2) 195.5(2), Ti(2)–O(13) 204.4(2), Ti(3)–O(1) 196.5(2), Ti(3)–O(19) 176.8(2), Ti(4)–O(6) 218.4(2), Ti(4)–O(9) 195.4(2), Ti(5)–O(4) 195.3(2), Ti(5)–O(6) 221.2(2), Ti(5)–O(12) 208.8(2), Ti(5)–O(22) 179.8(2), P(1)–O(2) 153.8(2), P(1)–O(4) 151.5(2), P(2)–O(6) 153.9(2), P(3)–O(9) 151.8(2); Ti(2)–O(1)–Ti(1) 105.18(8), Ti(4)–O(6)–Ti(5) 98.03(7)

The cluster Ti_5_(µ_3_–O)(µ_2_–O*i*Pr)_4_(O*i*Pr)_7_(OMc)(O_3_PEt)_3_ (**1**) was formed when bis(trimethylsilyl) ethylphosphonate was reacted with methacrylic acid (McOH) and Ti(O*i*Pr)_4_ in a 1:1:3 molar ratio (Fig. 1). This cluster type was previously obtained, as an exception from general outcome of the reactions with acetic acid mentioned in the “Introduction”, when bis(trimethylsilyl) 3-bromopropylphosphonate was reacted with acetic acid and Ti(O*i*Pr)_4_ in a 1:1:2 ratio at room temperature. The asymmetric unit of crystalline **1** contains two independent molecules with very similar bond distances and angles.

The structure of **1** is related to that of the clusters Ti_4_(µ_3_–O)(µ_2_–O*i*Pr)_3_(O*i*Pr)_5_(O_3_PR)_3_L (L = neutral ligand) [4–7], which consist of a symmetrical Ti_3_(μ_3_–O)(μ_2_-O*i*Pr)_3_(O*i*Pr)_3_ unit (Ti(1)–Ti(3) in Fig. 1 to which a Ti(O*i*Pr)_2_L group is connected by means of three phosphonate ligands. In **1**, the capping Ti(O*i*Pr)_2_L group is replaced by a Ti_2_(µ_2_–O*i*Pr)(O*i*Pr)_4_(µ_2_–OMc) moiety (Ti(4) and Ti(5) in Fig. 1). Two of the phosphonate ligands are coordinated to only one Ti atom of the Ti_2_ unit and have a 3.111 binding mode (w.xyz refers to the number of metal atoms to which the phosphonate ligand is coordinated [w], and the number of metal atoms to which each oxygen is coordinated [*x*, *y*, *z*] [8]), while the third bridges both of them and has a binding mode of 4.211. The degree of condensation of **1** is 0.2 (O/Ti ratio of the cluster core), while it is 0.67 for the clusters Ti_6_O_4_(O*i*Pr)_10_(OAc)_2_(O_3_PR)_2_ obtained with acetic acid under the same conditions. This indicates that ester + water formation of methacrylic acid, relative to the rate of substitution [9], is slower than that of acetic acid.

^1^H, ^13^C, and ^31^P NMR spectra of re-dissolved crystals of **1** in C_6_D_6_ showed numerous signals. In the ^31^P NMR spectrum, for example, eight resonances were observed, while two signals are expected if the solid-state structure of **1** was retained in solution. We therefore assume that **1** is in equilibrium with other compounds.

Isostructural Ti_5_(µ_3_–O)(µ_2_–O*i*Pr)_4_(O*i*Pr)_7_(OMc)(O_3_PCH_2_CH_2_CH_2_Br)_3_ (**2**) was obtained from the reaction of bis(trimethylsilyl) bromopropylphosphonate, methacrylic acid, and Ti(O*i*Pr)_4_ in a ratio of 1:2:3. The higher proportion of McOH thus did not influence the outcome of the reaction. Ti_2_(OMc)_2_(O*i*Pr)_6_*i*PrOH [8] was formed as a by-product, as proven by single crystal XRD. The ^1^H and ^31^P NMR spectra of the solid residue correspondingly showed numerous signals. Therefore, it can be assumed that a mixture of products was obtained and/or several species are in equilibrium with each other.

**Fig. 2:**
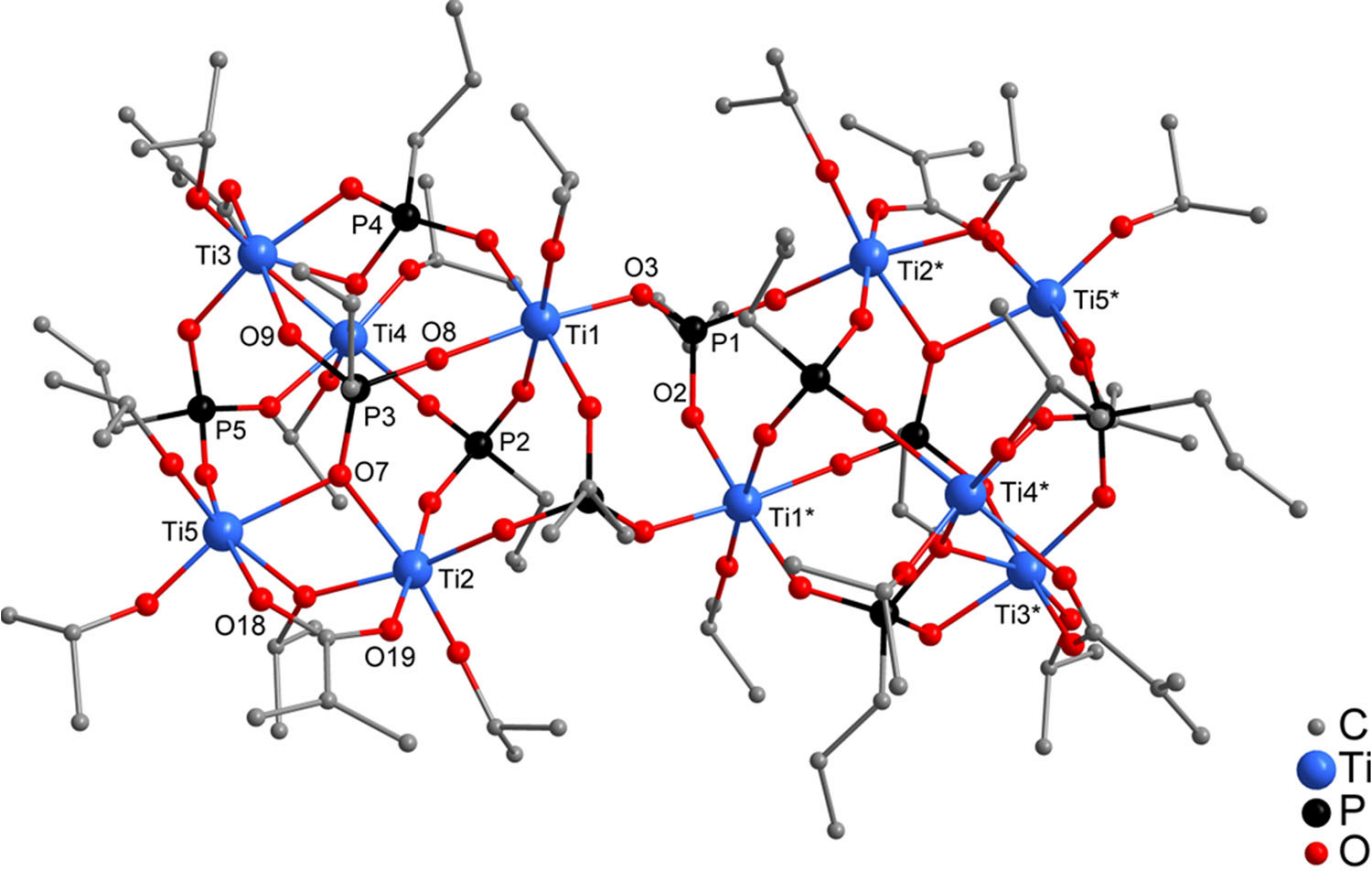
Molecular structure of Ti_10_(µ_2_–O*i*Pr)_2_(O*i*Pr)_14_(OMc)_4_(O_3_PCH_2_CH = CH_2_)_10_ (**3**). Hydrogen atoms are omitted for clarity. Selected bond lengths/pm and angles/°: Ti(1)–O(2) 193.6(2), Ti(1)–O(3) 195.9(2), Ti(1)–O(4) 202.1(2), Ti(1)–O(8) 195.8(2), Ti(1)–O(12) 200.9(2), Ti(1)–O(20) 175.6(2), Ti(2)–O(7) 216.5(2), Ti(2)–O(19) 206.3(3), Ti(3)–O(9) 193.5(2), Ti(3)–O(10) 221.1(2), Ti(4)–O(5) 193.1(2), Ti(5)–O(7) 217.4(2), Ti(5)–O(18) 206.8(3); Ti(2)–O(7)–Ti(5) 99.38(9), Ti(2)–O(21)–Ti(5) 110.3(1), Ti(3)–O(10)–Ti(4) 126.8(1), O(1)–P(1)–O(2) 110.7(1), O(1)–P(1)–C(1A) 107.3(2), O(10)–P(4)–O(11) 99.1(1), O(10)–P(4)–O(12) 114.6(1), O(11)–P(4)–O(12) 115.6(1)

When a 1:2:3 mixture of bis(trimethylsilyl) allylphosphonate, methacrylic acid, and Ti(O*i*Pr)_4_ was heated to reflux, the complex Ti_10_(µ_2_–O*i*Pr)_2_(O*i*Pr)_14_(OMc)_4_(O_3_PCH_2_CH=CH_2_)_10_ (**3**) (Fig. 2) was obtained after crystallization from CH_2_Cl_2_. It is noteworthy that **3** contains no oxo groups, but more O*i*Pr groups were substituted by OMc or O_3_PR ligands compared to **1** and **2**. The different outcome of this reaction, compared to **1** and **2**, may be due to the higher reaction temperature. We have previously shown that higher reaction temperatures favor substitution over ester formation [9].

The structure of **3** consists of two Ti_5_(O*i*Pr)_8_(OMc)_2_(O_3_P-allyl)_5_ units, which are bridged by two (3.111) phosphonate ligands. The Ti_5_ units are composed of methacrylate-bridged dimers Ti_2_(µ_2_–O*i*Pr)(O*i*Pr)_3_(OMc) (Ti(2), Ti(5)) and Ti_2_(O*i*Pr)_3_(OMc) (Ti(3), Ti(4)), respectively, which are connected through phosphonate ligands among each other as well as to the fifth titanium atom (Ti(1)). Each of the octahedrally coordinated titanium atoms is at least bound to two different phosphonate ligands; Ti(1) is coordinated by five different oxygen atoms of phosphonate ligands and one O*i*Pr ligand. The complexity of the structure of **3** is also reflected in the different binding modes of the phosphonate ligands, of which six are 3.111, two are 3.211, and two are 4.211.

**Fig. 3:**
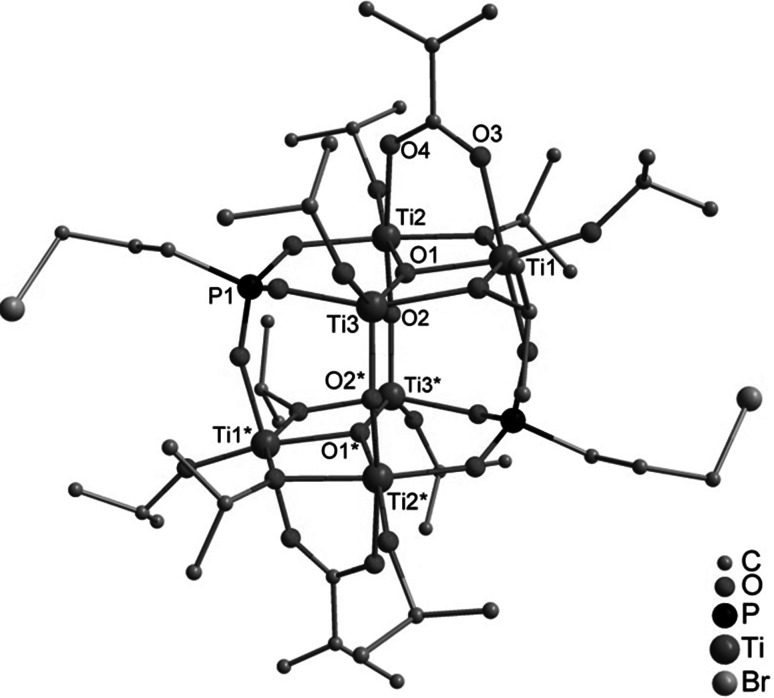
Molecular structure of Ti_6_O_4_(O*i*Pr)_10_(OMc)_2_(O_3_PCH_2_CH_2_CH_2_Br)_2_ (**4**). Hydrogen atoms are omitted for clarity. Selected bond lengths/pm and angles/°: Ti(1)–O(1) 197.1(1), Ti(1)–O(3) 208.8(1), Ti(1)–O(5) 199.0(1), Ti(1)–O(6) 196.1(1), Ti(1)–O(7) 177.7(1), Ti(1)–O(12) 192.9(1), Ti(2)–O(1) 199.2(1), Ti(2)–O(2) 187.3(1), Ti(2)–O(10) 196.5(1), Ti(3)–O(1) 189.9(1), Ti(3)–O(2)* 175.0(1), Ti(3)–O(6) 204.7(1), Ti(3)–O(9) 180.9(1); Ti(3)–O(1)–Ti(1) 105.83(6), Ti(3)–O(1)–Ti(2) 149.36(7), Ti(1)–O(1)–Ti(2) 104.15(6), Ti(3)–O(2)–Ti(2) 148.49(8), Ti(1)–O(5)–Ti(2) 102.48(6), Ti(1)–O(6)–Ti(3) 100.75(5)

The reactions leading to **1**, **2**, and **3** show that clusters with a noticeably lower degree of condensation were formed compared to analogous reactions with acetic acid [1]. This is most probably due to the lower reaction rate of ester formation between methacrylic acid and isopropyl alcohol compared to that of acetic acid [10]. This assumption was proven by the deliberate addition of water to the reaction mixture. Thus, when bis(trimethylsilyl) 3-bromopropylphosphonate, methacrylic acid, Ti(O*i*Pr)_4_, and water were reacted in a 1:1:3:2 ratio, the cluster Ti_6_O_4_(O*i*Pr)_10_(OMc)_2_(O_3_PCH_2_CH_2_CH_2_Br)_2_ (**4**) (Fig. 3) was obtained. The cluster **4** is isostructural to Ti_6_O_4_(O*i*Pr)_10_(OAc)_2_(O_3_PCH_2_CH_2_CH_2_Br)_2_ obtained with acetic acid [1].

The centrosymmetric cluster **4** is isostructural to the previously reported acetate-substituted clusters Ti_6_O_4_(O*i*Pr)_10_(OAc)_2_(O_3_PR)_2_ [1]. The cluster core is formed by two parallel, unsymmetrically substituted Ti_3_(µ_3_–O)(µ_2_–O*i*Pr)_2_(O*i*Pr)_3_(µ_2_–OMc) units connected by µ_2_-oxo (O(2) and O(2)*, * denotes symmetry-related atoms) and phosphonate bridges. Ti(1) and Ti(2) are bridged by both an O*i*Pr and a methacrylate ligand and are octahedrally coordinated while Ti(3) has a distorted trigonal bipyramidal coordination sphere. The central Ti_3_O unit is unsymmetrical, with one short (Ti(3)–O(1) 189.9(1) pm) and two long Ti–O distances (Ti(1)–O(1) 197.1(1), Ti(2)–O(1) 199.2(2) pm), as in the acetate derivatives Ti_6_O_4_(O*i*Pr)_10_(OAc)_2_(O_3_PR)_2_.

NMR data show that the structure of **4**, especially also their inversion symmetry is retained in solution. Thus, one signal was observed in the ^31^P NMR spectrum at 27.34 ppm. In the ^1^H NMR spectrum five doublets for the methyl groups of the O*i*Pr ligands were observed and three signals for the CH groups (at 4.86, 4.97, and 5.33 ppm) the latter two with double intensity. One singlet at 2.08 ppm and two multiplets at 5.41 and 6.36 ppm can be assigned to the two OMc ligands. In the ^13^C NMR spectrum only one doublet for each P-CH_2_ group was found and one set of signals for the OMc ligands. The signals of the O*i*Pr ligands were partly overlapping.

The clusters **5**–**12** with a great variety of functional or non-functional phosphonate ligands were obtained according to Scheme 1 by the same synthesis procedure as that for **4**. The ^1^H, ^31^P, and ^13^C NMR spectra of **5**–**12** are similar to that of **4**.

**Figure:**
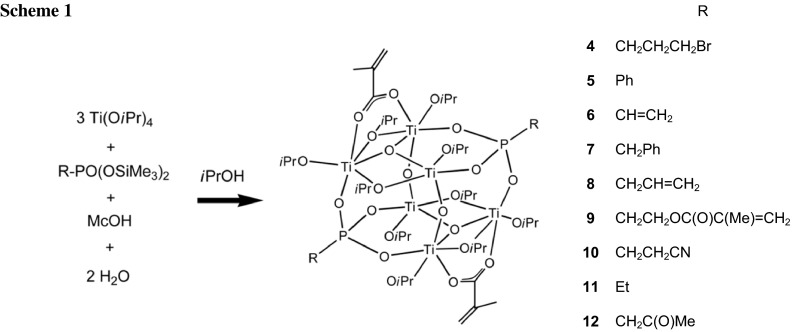

## Conclusions

The first step in reactions of metal alkoxides with carboxylic or phosphonic acids is the substitution of an OR ligand by a carboxylate or phosphonate ligand. The thus liberated alcohol can undergo ester formation with the carboxylic or phosphonic acid, which produces water that hydrolyzes part or all of the remaining M–OR groups. Thus two reactions, viz. substitution and ester formation, compete with each other, and their relative rate is one of the decisive parameters influencing the outcome of such reactions. How the clusters are formed from the initially formed M(OR)_*x*_(carboxylate/phosphonate)_*y*_ derivatives has not been elucidated in any case. The situation becomes even more complex when two different metal alkoxides or, as in the present case, two different acids are involved.

In previous work, we had preferentially obtained phosphonate/acetate-substituted titanium oxo/alkoxo clusters of the composition Ti_6_O_4_(O*i*Pr)_10_(OAc)_2_(O_3_PR)_2_ from Ti(O*i*Pr)_4_ and bis(trimethylsilyl) phosphonates in the presence of acetic acid (AcOH) [1]. The results of the work reported in this article show that the degree of condensation of the obtained clusters (**1** and **2**) was lower when acetic acid was replaced by methacrylic acid. In one case, the product (compound **3**) contained no oxo groups at all. This can be taken as evidence that the rate of esterification of methacrylic acid is lower than that of acetic acid.

The lower esterification rate can be compensated, however, by controlled addition of a stoichiometric amount of “external” water. The thus obtained methacrylate/phosphonate-substituted clusters **4**–**12**, with a very wide variety of phosphonate ligands, are isostructural to the acetate-substituted clusters Ti_6_O_4_(O*i*Pr)_10_(OAc)_2_(O_3_PR)_2_ obtained in earlier experiments [1]. Incorporation of the polymerizable OMc ligands is a very interesting option for the preparation of cluster-crosslinked polymers [2, 3], especially because this allows the combination (a) of reactive and non-reactive ligands as well as (b) ligands with different organic functionalities in a controlled manner in one cluster.

## Experimental

All operations were carried out in a moisture- and oxygen-free argon atmosphere using Schlenk techniques. Isopropyl alcohol was dried by refluxing twice over sodium metal and distillation. The bis(trimethylsilyl) phosphonates were prepared as reported before [1].

### Methacrylate-phoshonate-substituted Ti_5_ oxo clusters

Ti_5_O(OiPr)_11_(OMc)(O_3_PEt)_3_ (**1**): 1.6 cm^3^ of Ti(O*i*Pr)_4_ (5.42 mmol) was added to a solution of 500 mm^3^ of bis(trimethylsilyl) ethylphosphonate (1.81 mmol) and 153 mm^3^ of methacrylic acid (1.81 mmol) in 2 cm^3^ of isopropyl alcohol. Crystals of **1** were obtained from this solution after 8 weeks. Yield 410 mg (35 %).

Ti_5_O(OiPr)_11_(OMc)(O_3_PCH_2_CH_2_CH_2_Br)_3_ (**2**): 1.2 cm^3^ of Ti(O*i*Pr)_4_ (4.1 mmol) was added to a solution of 400 mm^3^ of bis(trimethylsilyl) 3-bromopropylphosphonate (1.34 mmol) in 2 cm^3^ of *i*PrOH, followed by addition of 113 mm^3^ of methacrylic acid (1.34 mmol). Crystals of **2** were obtained from this solution after 3 weeks. Yield 620 mg (mixture of compounds).

### Methacrylate-phoshonate-substituted Ti_10_ oxo cluster Ti_10_(OiPr)_16_(OMc)_4_(O_3_PCH_2_CHCH_2_)_10_ (**3**):

7.1 cm^3^ of Ti(O*i*Pr)_4_ (24 mmol) was added to a solution of 2 cm^3^ of bis(trimethylsilyl) allylphosphonate (8 mmol) and 1.35 cm^3^ of methacrylic acid (16 mmol) in 12 cm^3^ of isopropyl alcohol. The solution was heated to reflux for 16 h, and a suspension was formed. The solid was separated by filtration and recrystallized from CH_2_Cl_2_. Yield 120 mg (5 %). The crystals could not be re-dissolved in CD_2_Cl_2_ or another non-coordinating organic solvent and therefore no NMR measurements were performed.

#### Methacrylate-phoshonate-substituted Ti_6_ oxo clusters: Ti_6_O_4_(OiPr)_10_(OMc)_2_(O_3_PCH_2_CH_2_CH_2_Br)_2_ (**4**) and Ti_6_O_4_(OiPr)_10_(OMc)_2_(O_3_PPh)_2_ (**5**)

Ti(O*i*Pr)_4_ (1.17 cm^3^, 4 mmol) was quickly added to a solution of 400 mm^3^ of bis(trimethylsilyl) 3-bromopropylphosphonate (1.3 mmol) [or 225 mg of bis(trimethylsilyl) phenylphosphonate (0.82 mmol)] in 2 cm^3^ of 2-propanol followed by addition of 110 mm^3^ of methacrylic acid (1.3 mmol). Finally, 48 mm^3^ of water (2.7 mmol) diluted in 1 cm^3^ of 2-propanol was injected quickly directly into the solution. Crystals were obtained after 1 week.

**4**: yield 420 mg (43 %); ^1^H NMR (C_6_D_6_, 250 MHz): *δ* = 1.31 (d, ^3^*J*_H,H_ = 6.09 Hz, 12H, CH*Me*), 1.41 (d, ^3^*J*_H,H_ = 6.24 Hz, 12H, CH*Me*), 1.48 (d, ^3^*J*_H,H_ = 6.09 Hz, 12H, CH*Me*), 1.74 (d, ^3^*J*_H,H_ = 6.24 Hz, 12H, CH*Me*), 1.82 (d, ^3^*J*_H,H_ = 6.24 Hz, 12H, CH*Me*), 1.72–1.88 (m, 2H, PCH_2_), 2.08 (s, 6H, =CCH_3_), 2.36 (m, ^3^*J*_P,H_ = 15.84 Hz, ^3^*J*_H,H_ = 7.31 Hz, 2H, *CH*_*2*_CH_2_P), 3.46 (t, ^3^*J*_H,H_ = 7.16 Hz, CH_2_Br), 4.86 (m, ^3^*J*_H,H_ = 6.17 Hz, 2H, OCH), 4.97 (m, ^3^*J*_H,H_ = 6.13 Hz, 4H, OCH), 5.33 (m, ^3^*J*_H,H_ = 6.20 Hz, 4H, OCH), 5.39–5.43 (m, 2H, =CH_2_), 6.34–6.38 (m, 2H, =CH_2_) ppm; ^31^P NMR (C_6_D_6_, 101.2 MHz): *δ* = 27.34 ppm; ^13^C NMR (C_6_D_6_, 62.9 MHz): *δ* = 18.63 (CH*Me*), 23.89 (CH*Me*), 24.20 (CH*Me*), 24.81 (CH*Me*), 25.20 (CH*Me*), 25.67 (d, ^1^*J*_P,C_ = 157 Hz, PCH_2_), 27.68 (d, ^2^*J*_P,C_ = 4.53 Hz, *CH*_*2*_CH_2_P), 33.81 (d, ^3^*J*_P,C_ = 14.96 Hz, CH_2_Br), 77.83 (OCH), 78.69 (OCH), 79.40 (OCH), 123.41 (=CH_2_), 140.02 (=*C*Me–), 173.39 (COO) ppm.

**5**: yield 160 mg (27 %); ^1^H NMR (C_6_D_6_, 250 MHz): *δ* = 1.26 (d, ^3^*J*_H,H_ = 6.13 Hz, 12H, CH*Me*), 1.43 (d, ^3^*J*_H,H_ = 6.08 Hz, 24H, CH*Me*), 1.82 (d, ^3^*J*_H,H_ = 6.40 Hz, 12H, CH*Me*), 1.84 (d, ^3^*J*_H,H_ = 6.40 Hz, 12H, CH*Me*), 2.14 (s, 6H, CH_3_ (OMc)), 4.88 (m, ^3^*J*_H,H_ = 6.17 Hz, 2H, CH (O*i*Pr)), 4.99 (m, ^3^*J*_H,H_ = 6.13 Hz, 4H, CH (O*i*Pr)), 5.36–5.58 (m, 6H, CH (O*i*Pr) + CH_2_ (OMc)), 6.43–6.46 (m, 2H, CH_2_ (OMc)), 7.10–7.28 (m, 2H, CH (Ph)), 7.34–7.44 (m, 4H, Ph), 8.33–8.44 (m, 4H, Ph) ppm; ^31^P NMR (C_6_D_6_, 101.2 MHz): *δ* = 16.03 ppm; ^13^C NMR (C_6_D_6_, 62.9 MHz): *δ* = 18.64 (CH*Me*), 24.06 (CH*Me*), 24.26 (CH*Me*), 24.83 (CH*Me*), 25.17 (CH*Me*), 77.93 (OCH), 78.78 (OCH), 79.48 (OCH), 123.28 (=CH_2_), 130.25 (d, *J*_P,C_ = 2.99 Hz, Ph), 130.99 (d, *J*_P,C_ = 9.36 Hz, Ph), 131.80 (d, *J*_P,C_ = 9.97 Hz, Ph), 134.50 (d, ^1^*J*_P,C_ = 208.43 Hz, Ph), 140.25 (=*C*Me–), 173.46 (COO) ppm.

*Ti*_*6*_*O*_*4*_*(OiPr)*_*10*_*(OMc)*_*2*_*(O*_*3*_*P*–*CH*=*CH*_*2*_*)*_*2*_*(****6****), Ti*_*6*_*O*_*4*_*(OiPr)*_*10*_*(OMc)*_*2*_*(O*_*3*_*PCH*_*2*_*Ph)*_*2*_*(****7****), Ti*_*6*_*O*_*4*_*(OiPr)*_*10*_*(OMc)*_*2*_*(O*_*3*_*PCH*_*2*_–*CH*=*CH*_*2*_*)*_*2*_*(****8****), Ti*_*6*_*O*_*4*_*(OiPr)*_*10*_*(OMc)*_*2*_*(O*_*3*_*PCH*_*2*_*CH*_*2*_–*OMc)*_*2*_*(****9****), Ti*_*6*_*O*_*4*_*(OiPr)*_*10*_*(OMc)*_*2*_*(O*_*3*_*PCH*_*2*_*CH*_*2*_*C≡N)*_*2*_*(****10****), Ti*_*6*_*O*_*4*_*(OiPr)*_*10*_*(OMc)*_*2*_*(O*_*3*_*PCH*_*2*_*CH*_*3*_*)*_*2*_*(****11****), Ti*_*6*_*O*_*4*_*(OiPr)*_*10*_*(OMc)*_*2*_*(O*_*3*_*PCH*_*2*_*COCH*_*3*_*)*_*2*_*(****12****)*.

Compared to **4** and **5**, the synthesis was slightly modified. In the synthesis of **6** 660 mm^3^ of Ti(O*i*Pr)_4_ (2.27 mmol) was added to a mixture of 200 mm^3^ of bis(trimethyl)silyl vinylphosphonate (0.76 mmol) and 64 mm^3^ of methacrylic acid (0.76 mmol) in 2 cm^3^ of 2-propanol. Immediately afterwards, 27.3 mm^3^ of water (1.52 mmol) diluted in 0.5 cm^3^ of 2-propanol was added. Crystals of **6** were obtained after 3 days. The syntheses of **7**–**11** were analogous. The synthesis of **12** was done analogously, but the precursor solution was additionally heated after addition of water until a clear solution was obtained.

**6**: yield 70 mg (14 %); ^1^H NMR (C_6_D_6_, 250 MHz): *δ* = 1.33 (d, ^3^*J*_H,H_ = 6.09 Hz, 12H, OCH*Me*), 1.41 (d, ^3^*J*_H,H_ = 6.09 Hz, 12H, OCH*Me*), 1.51 (d, ^3^*J*_H,H_ = 5.94 Hz, 12H, OCH*Me*), 1.79 (d, ^3^*J*_H,H_ = 6.24 Hz, 12H, OCH*Me*), 1.84 (d, ^3^*J*_H,H_ = 6.09 Hz, 12H, OCH*Me*), 2.09 (s, 6H, =CMe), 4.87 (m, ^3^*J*_H,H_ = 6.13 Hz, 2H, OCH), 5.02 (m, ^3^*J*_H,H_ = 5.90 Hz, 4H, OCH), 5.30–5.45 (m, 6H, OCH + =CH_2_), 5.74 (ddd, ^2^*J*_H,H_ = 3.50 Hz, ^3^*J*_H,H_ = 12.03 Hz, ^3^*J*_P,H_ = 49.42 Hz, 4H, OCH), 6.21–6.57 (m, 6H, CH_2_(vinyl) + CH(vinyl) + =CH_2_ (OMc)) ppm; ^31^P NMR (C_6_D_6_, 101.2 MHz): *δ* = 14.25 ppm; ^13^C NMR (C_6_D_6_, 62.9 MHz): *δ* = 18.58 (=CMe), 23.93 (OCH*Me*), 24.23 (OCH*Me*), 24.78 (OCH*Me*), 25.17 (OCH*Me*), 77.82 (OCH), 78.65 (OCH), 79.38 (OCH), 123.16 (=CH_2_ (OMc), 128.66 (=CH_2_ (vinyl)), 130.81 (d (^1^*J*_P,C_ = 204.2 Hz), CH (vinyl)), 140.22 (=*C*Me), 173.41 (COO) ppm.

**7**: yield 160 mg (34 %); ^1^H NMR (C_6_D_6_, 250 MHz): *δ* = 1.30 (d, ^3^*J*_H,H_ = 6.09 Hz, 12H, OCH*Me*), 1.41–1.49 (m, 24H, OCH*Me*), 1.65 (d, ^3^*J*_H,H_ = 6.24 Hz, 12H, OCH*Me*), 1.72 (d, ^3^*J*_H,H_ = 6.24 Hz, 12H, OCH*Me*), 2.07 (s, 6H, = CMe), 3.20 (d, ^2^*J*_P,H_ = 22.69 Hz, 4H, PCH_2_), 4.83–5.02 (m, ^3^*J*_H,H_ = 6.07 Hz, 6H, OCH), 5.18–5.33 (m, ^3^*J*_H,H_ = 6.24 Hz, 6H, OCH), 5.40 (br, 2H, = CH_2_), 6.32 (br, 2H, = CH_2_), 7.18–7.28 (m, 2H, Ph), 7.31–7.39 (m, 4H, Ph), 7.63–7.68 (m, 4H, Ph) ppm; ^31^P NMR (C_6_D_6_, 101.2 MHz): *δ* = 23.34 ppm; ^13^C NMR (C_6_D_6_, 62.9 MHz): *δ* = 18.64 (=CMe), 23.81 (OCH*Me*), 24.15 (OCH*Me*), 24.95 (OCH*Me*), 25.26 (OCH*Me*), 35.18 (d, ^1^*J*_P,C_ = 152.25 Hz, PCH_2_), 77.74 (OCH), 78.69 (OCH), 79.05 (OCH), 123.12 (CH_2_ (OMc), 125.83 (d, *J*_P,C_ = 3.00 Hz, Ph), 130.44 (d, *J*_P,C_ = 6.98 Hz, Ph), 134.88 (d, *J*_P,C_ = 8.98 Hz, Ph), 140.19 (=CMe–), 173.34 (COO) ppm.

**8**: yield 180 mg (33 %); ^1^H NMR (C_6_D_6_, 250 MHz): *δ* = 1.31 (d, ^3^*J*_H,H_ = 6.10 Hz, 12H, OCH*Me*), 1.41 (d, ^3^*J*_H,H_ = 6.10 Hz, 12H, OCH*Me*), 1.50 (d, ^3^*J*_H,H_ = 6.03 Hz, 12H, OCH*Me*), 1.77 (d, ^3^*J*_H,H_ = 6.24 Hz, 12H, OCH*Me*), 1.84 (d, ^3^*J*_H,H_ = 6.21 Hz, 12H, OCH*Me*), 2.09 (s, 6H, =CMe), 2.68 (dd, ^2^*J*_P,H_ = 22.69 Hz, ^3^*J*_H,H_ = 7.08 Hz, 4H, PCH_2_), 4.85 (m, ^3^*J*_H,H_ = 6.09 Hz, 2H, OCH), 5.00 (m, ^3^*J*_H,H_ = 6.05 Hz, 4H, OCH), 5.22–5.45 (m, 10H, OCH + CH_2_ (OMc) + CH_2_ (allyl)), 6.16–6.35 (m, 2H, CH (allyl)), 6.38 (br, 2H, = CH_2_ (OMc)) ppm; ^31^P NMR (C_6_D_6_, 101.2 MHz): *δ* = 24.16 ppm; ^13^C NMR (C_6_D_6_, 62.9 MHz): *δ* = 18.59 (=CMe), 23.95 (OCH*Me*), 24.21 (OCH*Me*), 24.75 (OCH*Me*), 25.17 (OCH*Me*), 33.31 (d, ^1^*J*_P,C_ = 154.33 Hz, PCH_2_), 77.70 (OCH), 78.59 (OCH), 79.31 (OCH), 117.29 (d, ^3^*J*_P,C_ = 14.96 Hz, = CH_2_), 123.14 (CH_2_ (OMc)), 130.96 (d, ^2^*J*_P,C_ = 10.97 Hz, =CH), 140.20 (C (OMc)), 173.28 (COO) ppm.

**9**: yield 100 mg (22 %); ^1^H NMR (C_6_D_6_, 250 MHz): *δ* = 1.31 (d, ^3^*J*_H,H_ = 6.10 Hz, 12H, OCH*Me*), 1.42 (d, ^3^*J*_H,H_ = 6.15 Hz, 12H, OCH*Me*), 1.50 (d, ^3^*J*_H,H_ = 6.08 Hz, 12H, OCH*Me*), 1.76 (d, ^3^*J*_H,H_ = 6.24 Hz, 12H, OCH*Me*), 1.82 (d, ^3^*J*_H,H_ = 6.20 Hz, 12H, OCH*Me*), 1.89 (s, 6H, CH_3_ (OMc ester)), 2.10 (s, 6H, =CMe), 2.33–2.50 (m, 4H, PCH_2_), 4.79–5.06 (m, 10H, OCH + CH_2_O), 5.26 (br, 2H, CH_2_ (OMc ester)), 5.36 (m, ^3^*J*_H,H_ = 6.24 Hz, 4H, OCH), 5.43 (br, 2H, =CH_2_), 6.20 (br, 2H, CH_2_ (OMc ester)), 6.38 (br, 2H, =CH_2_) ppm; ^31^P NMR (C_6_D_6_, 101.2 MHz): *δ* = 23.59 ppm; ^13^C NMR (C_6_D_6_, 62.9 MHz): *δ* = 18.03 (CH_3_ (OMc ester)), 18.60 (=CMe), 23.90 (OCH*Me*), 24.19 (OCH*Me*), 24.71 (OCH*Me*), 25.16 (OCH*Me*), 27.88 (d, ^1^*J*_P,C_ = 152.46 Hz, PCH_2_), 60.92 (d, ^2^*J*_P,C_ = 3.98 Hz, OCH_2_), 78.03 (OCH), 78.80 (OCH), 79.71 (OCH), 123.67 (CH_2_ (OMc), 124.64 (CH_2_ (OMc ester)), 136.68 (C (OMc ester)), 139.94 (=CMe–), 166.56 (COO (OMc ester)), 173.50 (COO) ppm.

**10**: yield 120 mg (24 %); ^1^H NMR (C_6_D_6_, 250 MHz): *δ* = 1.26 (d, ^3^*J*_H,H_ = 6.09 Hz, 12H, OCH*Me*), 1.38 (d, ^3^*J*_H,H_ = 6.24 Hz, 12H, OCH*Me*), 1.41 (d, ^3^*J*_H,H_ = 6.09 Hz, 12H, OCH*Me*), 1.67 (d, ^3^*J*_H,H_ = 6.24 Hz, 12H, OCH*Me*), 1.77 (d, ^3^*J*_H,H_ = 6.24 Hz, 12H, OCH*Me*), 1.80–1.89 (m, 4H, CH_2_CN), 2.02 (s, 6H, =CMe), 2.54–2.66 (m, 4H, PCH_2_), 4.79 (m, 2H, OCH), 4.89 (m, 4H, OCH), 5.29 (m, 4H, OCH), 5.36 (br, 2H, =CH_2_), 6.29 (br, 2H, = CH_2_) ppm; ^31^P NMR (C_6_D_6_, 101.2 MHz): *δ* = 24.16 ppm; ^13^C NMR (C_6_D_6_, 62.9 MHz): *δ* = 11.67 (d, ^2^*J*_P,C_ = 2.50 Hz, CH_*2*_CN), 18.46 (=CMe), 22.04 (PCH_2_), 23.80 (OCH*Me*), 24.16 (OCH*Me*), 24.63 (OCH*Me*), 25.05 (OCH*Me*), 25.11 (OCH*Me*), 78.13 (OCH), 78.94 (OCH), 79.96 (OCH), 118.86 (d, ^3^*J*_P,C_ = 18.95 Hz, CN), 123.61 (=CH_2_), 139.83 (=CMe–), 173.48 (COO) ppm.

**11**: yield 230 mg (48 %); ^1^H NMR (C_6_D_6_, 250 MHz): *δ* = 1.23–1.47 (m, 6H, *CH*_*3*_CH_2_P), 1.32 (d, ^3^*J*_H,H_ = 6.09 Hz, 12H, OCH*Me*), 1.41 (d, ^3^*J*_H,H_ = 6.24 Hz, 12H, OCH*Me*), 1.50 (d, ^3^*J*_H,H_ = 6.09 Hz, 12H, OCH*Me*), 1.65–1.93 (m, 4H, PCH_2_), 1.77 (d, ^3^*J*_H,H_ = 6.24 Hz, 12H, OCH*Me*), 1.84 (d, ^3^*J*_H,H_ = 6.24 Hz, 12H, OCH*Me*), 2.09 (s, 6H, =CMe), 4.86 (m, ^3^*J*_H,H_ = 6.09 Hz, 2H, OCH), 5.00 (m, ^3^*J*_H,H_ = 6.09 Hz, 4H, OCH), 5.27–5.37 (m, 4H, OCH), 5.39 (br, 2H, CH_2_ (OMc)), 6.36 (br, 2H, CH_2_ (OMc)) ppm; ^31^P NMR (C_6_D_6_, 101.2 MHz): *δ* = 29.82 ppm; ^13^C NMR (C_6_D_6_, 62.9 MHz): *δ* = 7.42 (d, ^2^*J*_P,C_ = 6.28 Hz, *CH*_*3*_CH_2_P), 18.58 (=CMe), 20.00 (d, ^1^*J*_P,C_ = 158.57 Hz, PCH_2_), 23.93 (OCH*Me*), 24.20 (OCH*Me*), 24.75 (OCH*Me*), 25.13 (OCH*Me*), 25.21 (OCH*Me*), 77.51 (OCH), 78.44 (OCH), 79.09 (OCH), 122.99 (=CH_2_), 140.28 (=CMe–), 173.23 (COO) ppm.

**12**: yield 260 mg (53 %); ^1^H NMR (C_6_D_6_, 250 MHz): *δ* = 1.32 (d, ^3^*J*_H,H_ = 6.09 Hz, 12H, OCH*Me*), 1.39 (d, ^3^*J*_H,H_ = 6.24 Hz, 12H, OCH*Me*), 1.48 (d, ^3^*J*_H,H_ = 6.09 Hz, 12H, OCH*Me*), 1.71 (d, ^3^*J*_H,H_ = 6.24 Hz, 12H, OCH*Me*), 1.80 (d, ^3^*J*_H,H_ = 6.24 Hz, 12H, OCH*Me*), 2.05 (s, 6H, =CMe), 2.46 (m, 4H, MeCO), 3.00 (d, ^2^*J*_P,H_ = 23.91 Hz, 4H, PCH_2_), 4.81 (m, ^3^*J*_H,H_ = 6.17 Hz, 2H, OCH), 4.98 (m, ^3^*J*_H,H_ = 6.13 Hz, 4H, OCH), 5.31 (m, ^3^*J*_H,H_ = 6.32 Hz, 4H, OCH), 5.38 (br, 2H, CH_2_ (OMc)), 6.32 (br, 2H, =CH_2_) ppm; ^31^P NMR (C_6_D_6_, 101.2 MHz): *δ* = 18.69 ppm; ^13^C NMR (C_6_D_6_, 62.9 MHz): *δ* = 18.51 (=CMe), 23.80 (OCH*Me*), 24.19 (OCH*Me*), 24.72 (OCH*Me*), 25.20 (OCH*Me*), 30.50 (*CH*_*3*_–CO), 45.25 (d, ^1^*J*_P,C_ = 139.17 Hz, PCH_2_), 78.29 (OCH), 78.94 (OCH), 79.90 (OCH), 123.59 (CH_2_ (OMc)), 139.89 (C (OMc)), 173.48 (COO), 199.11 (d, ^2^*J*_P,C_ = 5.58 Hz, C=O) ppm.

### X-ray structure analyses

All measurements were performed using Mo*K*_*α*_ radiation (*λ* = 71.073 pm). Data were collected on a Bruker Axs Smart Apex II four-circle diffractometer with *κ*-geometry at 100 K with *φ* and *ω*-scans and 0.5° frame width (Table 1) and corrected for polarization and Lorentz effects. An empirical absorption correction (Sadabs) was applied. The cell dimensions were refined with all unique reflections. Saint Plus (Bruker Analytical X-ray Instruments, 2007) was used to integrate the frames. Symmetry was checked with the program Platon.

**Table 1:** Crystal data and structure refinement details

Compound	**1**	**2**	**3**	**4**
Emp. formula	C_43_H_97_O_23_P_3_Ti_5_	C_46_H_100_Br_3_O_23_P_3_Ti_5_	C_94_H_182_O_54_P_10_Ti_10_	C_44_H_92_Br_2_O_24_P_2_Ti_6_
*M*_*r*_	1314.62	1593.4	2965.1	1514.35
Crystal system	Monoclinic	Monoclinic	Triclinic	Monoclinic
Space group	*P*2_1_/*c*	*Pc*	*P* $\bar{1}$	*P*2_1_/*n*
*a*/pm	2756.6 (1)	1291.63 (5)	1366.83 (13)	1346.04 (2)
*b*/pm	1861.8 (1)	2301.65 (9)	1403.15 (13)	1532.03 (3)
*c*/pm	2600.8 (1)	2441.36 (9)	2110.25 (17)	1689.82 (3)
*α*/°	90	90	73.525 (4)	90
*β*/°	105.268 (2)	101.4782 (14)	75.199 (4)	108.9870 (10)
*γ*/°	90	90	64.922 (5)	90
*V*/pm^3^ × 10^6^	12877 (1)	7112.7 (5)	3472.3 (5)	3295.11 (10)
*Z*	8	4	1	2
*D*_*x*_/g cm^−3^	1.36	1.488	1.418	1.526
*µ*/mm^−1^	0.735	2.355	0.739	2.023
Crystal size/mm	0.25 × 0.2 × 0.15	0.52 × 0.15 × 0.1	0.25 × 0.15 × 0.1	0.48 × 0.44 × 0.4
No. measured refl.	230,762	101,719	76,823	43500
Obs. refl. [*I* > 2*σ* (*I*)]	17,441	23,566	8533	8180
*θ*_max_/°	25.07	26.37	25.15	30.55
R [*F* ^2^ > 2*σ*(*F*)], w*R* (*F* ^2^), *S*	0.0355, 0.0965, 1.074	0.0433, 0.1134, 1.076	0.0429, 0.1083, 1.035	0.0386, 0.1101, 1.089
Refl./param.	22815/1413	27296/1497	12361/828	10086/393
Weighting scheme^a^	*a* = 0.0409P, *b* = 14.5476	*a* = 0.0588, *b* = 8.0538	*a* = 0.0457, *b* = 3.3285	*a* = 0.0621, *b* = 0.1815
δ*ρ*_max_, _min_/e × 10^−6^ pm^−3^	1.00, −0.92	1.418, −1.716	0.78, −0.49	1.329, −1.132

Compound	**5**	**6**	**7**	**8**
Emp. formula	C_50_H_90_O_24_P_2_Ti_6_	C_42_H_84_O_24_P_2_Ti_6_	C_52_H_94_O_24_P_2_Ti_6_	C_44_H_90_O_24_P_2_Ti_6_
*M*_r_	1424.56	1322.43	1452.61	1352.5
Crystal system	Monoclinic	Triclinic	Monoclinic	Triclinic
Space group	*P*2_1_/*n*	*P* $\bar{1}$	*P*2_1_/*n*	*P* $\bar{1}$
*a*/pm	1388.4 (1)	1162.8 (1)	1376.21 (9)	1270.28 (9)
*b*/pm	1742.4 (2)	1264.4 (2)	1326.97 (8)	1443.4 (1)
*c*/pm	1411.0 (1)	1265.7 (2)	1936.70 (11)	1838.2 (1)
*α*/°	90	107.721 (3)	90	92.141 (3)
*β*/°	91.423 (3)	95.875 (3)	101.120 (2)	90.567 (3)
*γ*/°	90	113.308 (3)	90	94.006 (3)
*V*/pm^3^ × 10^6^	3412.4 (5)	1574.4 (3)	3470.4 (4)	3359.7 (4)
*Z*	2	1	2	2
*D*_*x*_/g cm^−3^	1.386	1.395	1.39	1.337
*µ*/mm^−1^	0.785	0.844	0.773	0.793
Crystal size/mm	0.45 × 0.42 × 0.38	0.42 × 0.38 × 0.37	0.38 × 0.37 × 0.3	0.42 × 0.37 × 0.34
No. measured refl.	86,933	37,906	42,971	81,110
Obs. refl. [*I* > 2*σ* (*I*)]	5075	3906	9040	12,077
*θ*_max_/°	26.4	25.14	30.51	28.6
R [*F* ^2^ > 2*σ*(*F*)], w*R* (*F* ^2^), *S*	0.0869, 0.2767, 1.171	0.0928, 0.2097, 1.059	0.0322, 0.1005, 1.088	0.0588, 0.1819, 1.075
Refl./param.	6991/382	5595/413	10,593/495	16,783/799
Weighting scheme^a^	*a* = 0.1198, *b* = 22.3579	*a* = 0.0146, *b* = 20.0056	*a* = 0.0503, *b* = 2.2610	*a* = 0.0765, *b* = 4.8222
δ*ρ*_max_, _min_/e × 10^−6^ pm^−3^	1.945, −0.978	1.541, −0.836	1.131, −0.82	1.317, −0.753

Compound	**9**	**10**	**11**	**12**
Emp. formula	C_50_H_98_O_28_P_2_Ti_6_	C_44_H_87_N_2_O_24_P_2_Ti_6_	C_42_H_90_O_24_P_2_Ti_6_	C_44_H_90_O_26_P_2_Ti_6_
*M*_r_	1496.62	1377.5	1328.48	1384.5
Crystal system	Triclinic	Monoclinic	Triclinic	Monoclinic
Space group	*P* $\bar{1}$	*P*2_1_/*c*	*P* $\bar{1}$	*C*2/*c*
*a*/pm	1316.93 (9)	1993.57 (3)	1191.1 (1)	2114.2 (6)
*b*/pm	1318.99 (9)	1909.15 (3)	1254.1 (1)	1306.8 (3)
*c*/pm	2275.93 (16)	1830.71 (3)	1321.9 (1)	2454.2 (7)
*α*/°	93.586 (2)	90	67.017 (3)	90
*β*/°	98.919 (2)	99.4200 (10)	89.871 (3)	104.112 (8)
*γ*/°	113.5566 (19)	90	65.009 (3)	90
*V*/pm^3^ × 10^6^	3546.1 (4)	6873.77 (19)	1616.0 (3)	6576 (3)
*Z*	2	4	1	4
*D*_*x*_/g cm^−3^	1.402	1.331	1.365	1.399
*µ*/mm^−1^	0.763	0.777	0.823	0.814
Crystal size/mm	0.45 × 0.43 × 0.4	0.48 × 0.42 × 0.38	0.55 × 0.5 × 0.45	0.51 × 0.41 × 0.32
No. measured refl.	136,754	52,967	15,444	130,844
Obs. refl. [*I* > 2*σ* (*I*)]	17,747	8622	3838	7964
*θ*_max_/°	30.56	25.11	25.03	30.58
R [*F* ^2^ > 2*σ*(*F*)], w*R* (*F* ^2^), *S*	0.0567, 0.1295, 1.079	0.063, 0.1999, 1.074	0.0922, 0.2265, 1.045	0.067, 0.1946, 1.165
Refl./param.	21,716/894	12,226/724	5543/492	10,080/407
Weighting scheme^a^	*a* = 0.0178, *b* = 11.8092	*a* = 0.0994, *b* = 8.5071	*a* = 0.0483, b = 17.8591	*a* = 0.0659, b = 45.3501
δ*ρ*_*max*, min_/e × 10^−6^ pm^−3^	1.49, −1.873	0.732, −0.342	1.435, −0.834	1.147, −0.653

^a^ *w* = $$ \frac{1}{{\sigma^{2} (F_{0} )^{2} + (aP)^{2} + bP}} $$ where *P* = $$ \frac{{F_{0}^{2} + 2F_{\text{c}}^{2} }}{3} $$

The structures were solved by the Patterson method (Shelxs97). Refinement was performed by the full-matrix least-squares method based on *F*^*2*^ (Shelxl97) with anisotropic thermal parameters for all non-hydrogen atoms. Hydrogen atoms were inserted in calculated positions and refined riding with the corresponding atom. In **1**, **3**, **4**, **6**–**9**, **11**, and **12** O*i*Pr ligands were disordered. In **6** and **12** one O*i*Pr ligand was additionally refined for three different positions. In **6**, **9**, and **11** the methacrylate ligand was bridging either between Ti(1) and Ti(2) or between Ti(1) and Ti(3). Two allyl groups in **3** and one Br atom in **2** were also disordered.

CCDC-1027711 (for **1**), -1027712 (for **2**), -1027713 (for **3**), -1027714 (for **4**), -1027715 (for **5**), -1027716 (for **6**), -1027717 (for **7**), -1027718 (for **8**), -1027719 (for **9**), -1027720 (for **10**), -1027721 (for **11**), and -1027722 (for **12**) contain the supplementary crystallographic data. These data can be obtained free of charge from the Cambridge Crystallographic Data Centre via http://www.ccdc.cam.ac.uk/data_request/cif.

## References

[1] Czakler M, Artner C, Schubert U (2014) Eur J Inorg Chem 2038 10.1002/ejic.201400051 PMC4362471 25814832

[2] Schubert U (2001). Chem Mater.

[3] Schubert U (2011). Chem Soc Rev.

[4] Guerrero G, Mehring M, Mutin PH, Dahan F, Vioux A (1999). J Chem Soc Dalton Trans.

[5] Mehring M, Guerrero G, Dahan F, Mutin PH, Vioux A (2000). Inorg Chem.

[6] Chakraborty D, Chandrasekhar V, Bhattacharjee M, Krätzner R, Roesky HW, Noltemeyer M, Schmidt H (2000). Inorg Chem.

[7] Czakler M, Artner C, Schubert U (2013) Eur J Inorg Chem 5790 10.1002/ejic.201400051 PMC4362471 25814832

[8] Chandrasekhar V, Senapati T, Dey A, Hossain S (2011). Dalton Trans.

[9] Czakler M, Artner C, Schubert U (2014) Monatsh Chem. doi:10.1007/s00706-015-1443-6

[10] Newman MS (1950). J Am Chem Soc.

